# No evidence of direct activation of human neutrophil responses by multivalent prefusion trimeric SARS-CoV-2 Spike protein *ex vivo*

**DOI:** 10.1371/journal.pone.0332261

**Published:** 2025-10-29

**Authors:** Audray Fortin, Sandrine Huot, Elise Caron, Cynthia Laflamme, Amélie Pagliuzza, Nicolas Chomont, Caroline Gilbert, Baoshan Zhang, Peter D. Kwong, Marc Pouliot, Nathalie Grandvaux

**Affiliations:** 1 Centre de recherche du Centre Hospitalier de l’Université de Montréal (CRCHUM), Montreal, Quebec, Canada; 2 Division of Infectious and Immune Diseases, Centre de Recherche du Centre Hospitalier Universitaire de Québec-Université Laval, Quebec City, Canada; 3 Department of microbiology, Infectious disease and immunology, Faculty of Medicine, Université Laval, Quebec City, Canada; 4 Department of microbiology, Infectiology and immunology, Faculty of medicine, Université de Montréal, Montreal, Canada; 5 Vaccine Research Center, National Institute of Allergy and Infectious Diseases, National Institutes of Health, Bethesda, Maryland, United States of America; 6 Department of Biochemistry and Molecular Biophysics and Aaron Diamond Research Center, Columbia University, New York, New York, United States of America; 7 Department of biochemistry and molecular medicine, Faculty of medicine, Université de Montréal, Montreal, Canada; Indian Institute of Chemical Technology, INDIA

## Abstract

The SARS-CoV-2 Spike (S) protein is essential for viral entry and serves as the primary immunogen in most COVID-19 vaccines. While its role in adaptive immunity is well defined, its potential to contribute directly to innate immune activation remains incompletely understood. Neutrophils, in particular, are prominent effectors in COVID-19 severity, yet how they respond directly to the S protein presented in a multivalent format is unclear. Here, we investigated whether the S protein can directly activate human neutrophils *ex vivo* using two biologically relevant models: nanoparticles displaying multivalent stabilized prefusion trimeric S glycoprotein, and purified β-propiolactone-inactivated SARS-CoV-2 virions. Neutrophils were exposed to nanoparticles or inactivated virus, either alone or pre-coated with monoclonal or polyclonal anti-S antibodies. Nanoparticles displaying Respiratory Syncytial Virus (RSV) Fusion (F) protein and purified β-propiolactone-inactivated RSV served as comparators. Across all models and conditions tested, the S protein did not induce significant neutrophil responses. No consistent effects were observed on cell viability, surface marker expression, reactive oxygen species production, neutrophil extracellular trap formation, cytokine release, or inflammatory gene expression—even in the presence of anti-S antibodies mimicking immune complexes. Results with F-nanoparticles and inactivated RSV were similarly modest. These findings indicate that the trimeric prefusion S protein, whether displayed multivalently on nanoparticles or in the context of inactivated viral particles, is insufficient to trigger robust neutrophil activation. This work provides insight into the innate immune profile of the S protein and suggests that its use in vaccine platforms is unlikely to directly provoke neutrophil-mediated inflammatory responses.

## Introduction

The emergence of severe acute respiratory syndrome coronavirus 2 (SARS-CoV-2) led to a global pandemic, with profound health and economic impact. Despite extensive research, the mechanisms underlying the broad clinical spectrum of COVID-19 —from asymptomatic cases to severe disease with multi-organ failure and long-term complications—remain incompletely understood [1]. Severe cases are frequently associated with dysregulated inflammatory cytokine response, contributing to tissue damage and systemic complications [2,3]. In the post-acute phase, a significant proportion of individuals develop persistent symptoms — known as post-acute sequelae of COVID-19, PASC or Long COVID [4]— potentially linked to the existence of a persisting SARS-CoV-2 reservoir in some patients [5,6].

Neutrophils are central to the innate immune response, but can also promote oxidative stress, tissue damage and systemic inflammation when dysregulated [7]. In COVID-19, their role in disease severity is supported by increased blood neutrophil counts, distinct activation patterns, neutrophil-driven immunopathology in affected organs and elevated NETs correlating with worst outcomes [8–16]. Yet, the molecular triggers for neutrophil activation in COVID-19 remain incompletely understood.

Among the viral components implicated in disease pathogenesis, the SARS-CoV-2 Spike (S) glycoprotein is of particular significance. The S protein facilitates viral entry by binding to the angiotensin-converting enzyme 2 (ACE2) receptor on host cells [17]. In its functional prefusion state, S is a trimeric protein composed of S1 and S2 subunits, with cleavage at the furin site separating the two domains. S1 mediates receptor engagement, while S2 undergoes conformational changes to drive membrane fusion [18–20]. As the principal target of neutralizing antibodies, the SARS-CoV-2 S protein has been central to vaccine development, pursued through various strategies, including mRNA-based (BNT162b2, Pfizer-BioNTech; mRNA-1273, Moderna), adenoviral vector-based (AZD1222/ChAdOx1 nCoV-19, AstraZeneca; Ad26.COV2.S, Janssen; Sputnik V, Gamaleya), and protein subunit vaccines (NVX-CoV2373, Novavax). With the exception of AZD1222/ChAdOx1 and Sputnik V, these vaccines are designed to elicit immune responses against a stabilized prefusion form of S, incorporating stabilizing mutations to prevent structural conversion and thereby optimize antigenicity and immunogenicity [21]. Inactivated virus-based vaccines (CoronaVac, Sinovac; BBIBP-CorV, Sinopharm; BBV152, Bharat Biotech) also aim to present the native prefusion S as an immunogen; however, post-fusion conformations may arise during purification [21]. Despite the occurrence of rare adverse effects, including vaccine-induced immune thrombotic thrombocytopenia (VITT) associated with AZD1222/ChAdOx1 nCoV-19 and Ad26.COV2.S adenoviral vector vaccines, vaccines have demonstrated a strong benefit-risk profile, effectively preventing severe disease, reducing hospitalizations, and mitigating the global impact of COVID-19 [22,23].

Beyond its role in viral entry, emerging evidence suggests that the S protein itself may contribute to COVID-19 immunopathology. Multiple pieces of evidence support that S can induce microvascular dysfunction, disrupt the blood-brain barrier, and trigger inflammatory responses in microglia and other immune cells [24–26]. Furthermore, consistent with the presence of a SARS-CoV-2 reservoir, the S protein has been detected in tissues and circulation long after acute infection, suggesting a potential role in sustained inflammation and post-acute sequelae [27–29]. While rare, adverse effects of COVID-19 vaccines such as myocarditis and thrombotic events share similarities with severe COVID-19 complications. This has led to the speculation that the S protein detected in the plasma of vaccine recipients, particularly those with myocarditis, may contribute to inflammation-related adverse reactions [30–33]. Finally, while there is little clinical evidence of antibody-dependent enhancement of disease, SARS-CoV-2 S-specific IgG have been linked to excessive inflammatory responses in COVID-19 [34].

Despite previous research on neutrophils and the SARS-CoV-2 S protein, the functional interaction of the trimeric prefusion glycoprotein in a multivalent format remains poorly understood. While most available studies have used soluble recombinant S protein to assess neutrophil activation, here we investigated whether the S protein can directly activate human neutrophils *ex vivo* using two biologically relevant models: a stabilized prefusion trimeric S protein displayed multivalently on nanoparticles, and β-propiolactone-inactivated SARS-CoV-2 virions. Strikingly, our findings reveal little to no direct neutrophil activation in response to the S protein, challenging the notion that S is sufficient to trigger robust neutrophil activation.

## Materials and methods

### Materials

Dextran-500, Phorbol 12-myristate 13-acetate (PMA), Luminol, β-propiolactone (BPL) and PEG-6000 were purchased from Sigma-Aldrich (Oakville, ON, Canada). Lymphocyte separation medium was from Wisent (St-Bruno, QC, Canada). Lyophilized human immunoglobulins G (IgGs), purified from human plasma or serum by fractionation (purity >97% determined by SDS-PAGE), were from Innovative Research, Inc (Novi, MI, USA). Fc Block™ was from BD Biosciences (San Jose, CA, USA). SYTOX™ Green and CellTrace™ Violet were from Thermo Fisher Scientific (Burlington, ON, Canada).

### Nanoparticles displaying trimeric prefusion SARS-CoV-2 S or RSV-F protein

Self-assembling nanoparticles with a lumazine synthase (LuS)-based scaffold with multivalent display of viral glycoprotein used in this study were produced and validated as we previously described [35]. The LuS-N71-SpyLinked-RSV F nanoparticles (F-nanoparticles; M.W. 2584.2 kDa) display prefusion-stabilized trimeric respiratory syncytial virus (RSV) fusion (F) glycoprotein (DS2-preF stabilized RSV F). Similarly, the LuS-N71-SpyLinked-CoV-2 S nanoparticles (S-nanoparticles; M.W. 6260.1 kDa) present the prefusion-stabilized trimeric version (GSAS and PP mutations and the T4 phage fibritin trimerization domain) of the SARS-CoV-2 S protein.

### SARS-CoV-2 amplification and purification

All experiments involving SARS-CoV-2 culture were conducted in a certified containment level-3 facility following standard operating procedures approved by the Biosafety Committee at CRCHUM, Montreal, Canada. The SARS-CoV-2/SB2 isolate [36] was obtained from Dr. Samira Mubareka (Sunnybrook Research Institute, Toronto, Canada) and propagated in VERO E6 cells (ATCC; Research resource IDentification (RRID): CVCL_0574) as described in [37]. Viral stocks were sequenced and tested for mycoplasma contamination (Invivogen). SARS-CoV-2 was amplified in VERO E6 cells in DMEM (GIBCO) supplemented with 1% L-glutamine and 2% Fetaclone III (Hyclone) at a multiplicity of infection (MOI) of 0.02. At 96 h post-infection, the supernatant was harvested and clarified by centrifugation at 4000g for 15 min to remove cell debris. Viral inactivation was achieved by treatment with 0.05% (v/v) β-propiolactone (BPL) for 16 h at 4°C, followed by hydrolysis at 37°C for 2 h. Inactivation was confirmed by the absence of viral replication in VERO E6 cells, quantified using the median tissue culture infectious dose (TCID50) method [37]. The clarified inactivated supernatant was precipitated by gentle stirring on ice for 90 min with 10% PEG-6000 in the presence of 100 mM MgSO₄. The pellet was dissolved in cold NTE buffer (150 mM NaCl, 50 mM Tris-HCl pH 7.5, 1 mM EDTA) and further purified using a discontinuous sucrose gradient (10%, 20%, and 30% sucrose in NTE). Ultracentrifugation was performed at 41,000 rpm (211,400 g) for 60 min at 4°C using a TH-641 Swinging Bucket Rotor (Thermo Scientific™). The purified virus pellet was gently resuspended in a minimal volume of cold NTE buffer to obtain a high-concentration virus stock, which was aliquoted and stored at −80°C. Viral fractions were resolved by SDS-PAGE and immunoblotted as previously described [38], using anti-SARS-CoV-2 S (BEI Resources, NR-52947; RRID: not available) and anti-SARS-CoV-2 N (Novus, NB100−56576; RRID: AB_838838) antibodies. Virion quantification was performed via RT-qPCR. RNA extraction from virus preparations was carried out using the QIAamp Viral RNA Mini Kit (Qiagen), and the *SARS-CoV-2 N* gene was quantified by qPCR on a QuantStudio 5 instrument using TaqPath 1-Step Multiplex Master Mix No ROX (Applied Biosystems). The following primers and probe were used: N_Forward: 5’-CGTACTGCCACTAAAGCATACA-3’; N_Reverse: 5’-GCGGCCAATGTTTGTAATCAG-3’; N_P: 5’-AGACGTGGTCCAGAACAAACCCAA-3’. A standard curve was generated using *in vitro* transcribed RNA, and the quantity of SARS-CoV-2 particles per mL was extrapolated based on *N* gene quantification.

### RSV A2 amplification and purification

All experiments involving RSV culture were conducted in a certified containment level-2 facility following standard operating procedures approved by the Biosafety Committee at CRCHUM, Montreal, Canada. The initial stock of the RSV A2 strain was obtained from Advanced Biotechnologies Inc. Virus amplification was performed in HEp-2 cells (ATCC; RRID: CVCL_1906) cultured in DMEM (GIBCO) supplemented with 1% L-glutamine (GIBCO) and 2% Fetaclone III (Hyclone) at a multiplicity of infection (MOI) of 0.1, until 50% cytopathic effect was observed. Cells and culture medium were harvested by scraping, followed by centrifugation at 3200 g for 20 min at 4°C. The resulting pellet underwent three freeze-thaw cycles before a final centrifugation step. Supernatants were pooled and inactivated by treatment with 0.05% (v/v) β-propiolactone (BPL) for 16 h at 4°C, followed by hydrolysis at 37°C for 2 h. Inactivated RSV was then precipitated with 10% PEG-6000 as described in [39] and resuspended in 20% sucrose in NTE buffer using a Dounce homogenizer. The virus suspension was layered onto a 30% sucrose cushion in NTE and ultracentrifuged at 25,000 rpm (106,000 g) at 4°C for 1 h using a SW41 Ti rotor (Beckman Coulter). The resulting pellet was resuspended in 5% sucrose in NTE using a Dounce homogenizer, aliquoted, and stored at −80°C until use. Virus inactivation was confirmed by methylcellulose plaque assays, as previously described [40]. Purified RSV was resolved by SDS-PAGE and immunoblotted using anti-RSV antibodies (Chemicon, AB1128; RRID: AB_90477). Virion quantification (particles per mL) was determined based on RSV A2 *N* gene quantification by RT-qPCR. RNA extraction and qPCR conditions were as described for SARS-CoV-2, except that amplification of the *N* gene was performed on a Rotor-Gene 3000 Real-Time Thermal Cycler (Corbett Research) using the FastStart SYBR Green Kit (Roche) with the following primers: N_Forward: 5’-AGATCAACTTCTGTCATCCAGCAA-3’; N_Reverse: 5’-TTCTGCACATCATAATTAGGAGTATCAAT-3’.

### Hydrodynamic size measurement

The size and homogeneity of nanoparticles and inactivated-viral particles size was analyzed by dynamic light scattering (DLS) with a Zetasizer Nano ZS (Malvern Instruments) as previously described in [41]. Hydrodynamic diameter measurements were done in duplicate at room temperature.

### Coating of nanoparticles and viruses with antibodies

Where indicated, S- and F-nanoparticles were pre-coated with specific antibodies against the S- or F-proteins, respectively, for 30 min at 37°C. For S-nanoparticles, a neutralizing monoclonal anti-S antibody (Clone CV30; Absolute Antibody, Boston, USA) was used. In subset experiments, purified SARS-CoV-2 virions were coated either with CV30 alone (αS) or with a combination of five monoclonal antibodies (αS Mix), each targeting a distinct epitope on the SARS-CoV-2 S protein RBD domain (Leinco Technologies Inc., St. Louis, MO, USA). At least one of these antibodies possesses neutralizing activity. All anti-S antibodies were produced in HEK293 cells, which provide human-like glycosylation patterns [42]. For F-nanoparticles, Palivizumab (Synagis, AbbVie), a recombinant humanized monoclonal IgG1 anti-F antibody was used [43]. The details of the anti-S and anti-F antibodies used in this study are provided in **Table 1**.

**Table 1:** Monoclonal antibodies used in this study.

Antibodies^a^	Clone	Isotype	[µg/mL]	Cat. Num.	RRID^b^ Num.	Supplier
Anti-SARS-CoV-2 S (αS)	CV30	IgG_1_, κ*	125	Ab02019–10.0	Not available	Absolute Antibody (Boston, MA, USA)
Anti-SARS-CoV-2 S Mix (αS Mix)	2165	IgG_1_*	25	LT1900	AB_2893932	LeincoTechnologies, Inc. (St. Louis, MO, USA)
	2196	IgG_1_	25	LT8000	AB_2894018	
	2355	IgG_1_	25	LT5000	AB_2893962	
	2381	IgG_1_	25	LT4000	AB_2893955	
	2838	IgG_1_	25	LT3000	AB_2893948	
Palivizumab Anti-RSV F (αF)	N/A	IgG_1_, κ*	125	DIN 02438372	Not available	AbbVie Corporation, (St-Laurent, QC, CA)

* Neutralizing antibodies.

^a^ *All anti-S antibodies target the RBD.*

^b^Research resource IDentification.

### Human neutrophils isolation

All experiments involving human tissues received approval from the Research Ethics Committee of CHU de Québec-Université Laval (2021−5449). Written informed consent was obtained from all donors. Data collection and analysis were performed anonymously. Participants were recruited from August 19, 2021 to January 13, 2022. Neutrophils were isolated as previously described, with modifications [44]. Briefly, venous blood from healthy volunteers was collected in isocitrate anticoagulant solution and centrifuged at 250 g for 10 min to separate the platelet-rich plasma, which was discarded. Leukocytes were obtained following erythrocyte sedimentation in 2% Dextran-500. Neutrophils were then separated from other leukocytes by centrifugation through a 10 mL layer of lymphocyte separation medium. Residual erythrocytes were eliminated by a 20-second hypotonic lysis step. The final granulocyte preparation consisted of >95% neutrophils and <5% eosinophils, with monocyte contamination below 0.2%, as determined by esterase staining. Cell viability exceeded 98%, as assessed by trypan blue dye exclusion. All isolation steps were performed under sterile conditions at room temperature.

### Preparation of heat-aggregated (HA) IgGs

HA-IgGs were prepared as previously described [45], with modifications. Briefly, soluble aggregates were freshly generated each day by resuspending IgGs in HBSS at a concentration of 25 mg/mL, followed by heating at 63°C for 75 min.

### Neutrophil incubation with nanoparticles or inactivated viruses

Neutrophils were resuspended in Hank’s Balanced Salt Solution (HBSS) supplemented with 10 mM HEPES (pH 7.4) and 1.6 mM Ca^2^ ⁺ , but without Mg^2^ ⁺ . The nanoparticle-to-neutrophil and virus particle-to-neutrophil ratios are provided in each figure.

### Neutrophil viability assessment

Neutrophil viability was evaluated using the LIVE/DEAD™ Fixable Dead Cell Stain Kit (Thermo Fisher Scientific, Burlington, ON, Canada). Briefly, cell pellets were resuspended in 1 mL of HBSS containing 1 μL of LIVE/DEAD stain and incubated for 30 min. Samples were then centrifuged, resuspended in 400 μL of 1% paraformaldehyde, and analyzed by flow cytometry.

### Analysis of neutrophil surface marker expression

Following treatment, cell suspensions were centrifuged and resuspended in HBSS containing human Fc Block™ (BD Biosciences). Cells were incubated with the appropriate mix of fluorophore-conjugated antibodies for 30 min at 4°C in the dark. After incubation, cells were centrifuged, resuspended in 400 μL of 1% paraformaldehyde, and analyzed by flow cytometry using a FACS Canto II instrument with FACSDiva software (version 6.1.3, BD Biosciences). A detailed list of labeled mouse monoclonal antibodies targeting the surface markers CD15, CD46, CD55, CD59, CD64, CD93, CD32, CD16, CD62L, CD63, CD66b, and CD11b (all from BD Biosciences, San Jose, CA, USA) is provided in **Table 2**.

**Table 2:** List and description of mouse monoclonal antibodies used in flow cytometry to monitor surface markers.

Monitoring	Human Target	Isotype	Conjugate	Dilution	Cat. Num.	RRID^a^ Num	Supplier
Immune complex receptors	**CD16**	IgG_1_, κ	PerCP-Cy™5.5	1: 3000	560717	AB_1727434	BD Biosciences
	**CD32**	IgG_2b_, κ	PE	1: 50	550586	AB_393766	BD Biosciences
	**CD64**	IgG_1_, κ	V450	1: 100	561202	AB_10564066	BD Biosciences
Adhesion molecules	**CD11b**	IgG_1_, κ	APC	1: 100	301410	AB_2280647	Biolegend
	**CD15**	IgM, κ	PerCP-Cy™5.5	1: 100	560828	AB_10563612	BD Biosciences
	**CD62L**	IgG_1_, κ	V450	1: 100	560440	AB_1645579	BD Biosciences
Degranulation	**CD63**	IgG_1_, κ	PE	1: 50	556020	AB_396298	BD Biosciences
	**CD66b**	IgM, κ	FITC	1: 200	555724	AB_396067	BD Biosciences
Complement regulatory elements	**CD46**	IgG_2a_, κ	APC	1: 100	564253	AB_2738705	BD Biosciences
	**CD55**	IgG_2a_, κ	FITC	1: 100	555693	AB_396044	BD Biosciences
	**CD59**	IgG_2a_, κ	PE	1: 100	555764	AB_396104	BD Biosciences
	**CD93**	IgG_2b_, κ	FITC	1: 100	551531	AB_394233	BD Biosciences

^a^Research resource IDentification.

### Measurement of ROS production by neutrophils

ROS production was measured as previously described [46], with modifications. Briefly, neutrophils were resuspended at 1 × 10⁶ cells/mL in HBSS supplemented with 10% heat-inactivated fetal bovine serum (FBS) and 10 μM luminol. Neutrophils (200 μL) were seeded into 96-well microplates, treated as indicated, and stimulated with or without 1 mg/mL HA-IgGs. Suspensions were incubated at 37°C in an Infinite M1000 PRO microplate reader (Tecan, Morrisville, NC, USA) using i-control 2.0 software. Luminescence intensity was recorded every 5 min.

### Measurement of Neutrophil Extracellular Trap (NET)

NET production was assessed as previously described [47], with modifications. Briefly, neutrophils were resuspended at 2.5 × 10^5^ cells/mL in HBSS, aliquoted into white 96-well plates (200 μL/well) and allowed to settle for 30 min at 37°C. Cells were then treated as indicated and incubated at 37°C with 5% CO₂ for 4 h. SYTOX Green (5 μM final concentration), a cell-impermeable nucleic acid stain, was added, and NET production was quantified by measuring fluorescence at excitation/emission wavelengths of 504/523 nm.

### Neutrophil RNA Isolation and RT-qPCR Analysis

Total RNA was isolated from neutrophils using Trizol™ Reagent (Thermo Fisher Scientific) following the manufacturer’s protocol, with modifications. Briefly, 25 × 10⁶ neutrophils were homogenized in 1 mL of Trizol, and 200 μL of chloroform was added. After mixing, samples were centrifuged at 12,000 g for 15 min at 4°C. The upper aqueous phase was transferred to a tube containing an equal volume of isopropanol, mixed by inversion, and centrifuged at 12,000g for 10 min at 4°C. The resulting RNA pellets were washed with 1 mL of 75% ethanol, followed by centrifugation at 10,000 g for 5 min at 4°C. After discarding the supernatants, pellets were air-dried for 10–15 min before resuspension in DEPC-treated water. RNA concentration was determined using a Qubit® Fluorometer (Thermo Fisher Scientific).

First-strand cDNA synthesis was performed using 1 µg of total RNA with the SuperScript™ II Reverse Transcriptase (Thermo Fisher Scientific), following the manufacturer’s instructions. Real-time PCR was carried out as described in [48] using SensiFAST SYBR® Lo-ROX mix (FroggaBio, Toronto, ON, Canada) in a Rotor-Gene Q system with Q-series software version 2.0.2 (Qiagen Inc., Mississauga, ON, Canada). Each reaction mixture contained 40 ng of cDNA and 500 nM primers in a final volume of 20 µL. Reaction specificity was confirmed using a melt curve procedure (58–99°C, 1°C per 5 seconds) at the end of the amplification protocol, according to the manufacturer’s instructions. Specific primers for each gene of interest were designed as previously described [49] and are listed in **Table 3**.

**Table 3:** Sequence of PCR primers used for neutrophils mRNA expression analyses.

Gene	Primers
GAPDH	**F**^a^: CGAGATCCCTCCAAAATCAA **R**^b^: TTCACACCCATGACGAACAT
	
CCL3 (MIP1α)	**F**: CTCTGCAACCAGGTCCTCTC **R**: TTTCTGGACCCACTCCTCAC
	
COX2	**F**: TGCATTCTTTGCCCAGCACT **R**: AAAGGCGCAGTTTACGCTGT
	
CXCL8 (IL-8)	**F**: GTGCAGTTTTGCCAAGGAGT **R**: CTCTGCACCCAGTTTTCCTT
	
IFNB	**F**: GAACTTTGACATCCCTGAGGAGATTAAGCAGC **R**: GTTCCTTAGGATTTCCACTCTGACTATGGTCC
	

^a^F: forward.

^b^R: reverse.

### Quantification of cytokine and chemokine release from neutrophils

Cell-free supernatants were stored at −20°C until analysis of cytokine and chemokine content using a multiplexed bead-based immunoassay (BD™ Cytometric Bead Array), following the manufacturer’s protocol. Measurements were acquired on a FACS Canto II flow cytometer and analyzed using FCAP Array software (version 3.0, BD Biosciences). The levels of IL-1α, IL-1β, IL-6, CXCL8 (IL-8), TNF, IFNα, CCL3 (MIP-1α), and CCL2 (MCP-1) were quantified.

### Statistical analysis

Statistical analysis was performed using GraphPad PRISM version 9 (GraphPad Software, San Diego, CA, USA). Where applicable, values are expressed as the mean ± standard error of the mean (SEM). Statistical analysis was performed using the Friedman test followed by a Dunn’s multiple comparisons post hoc test, unless stated otherwise. * *P* < 0.05; ** *P* < 0.01. Unless otherwise stated in the results section and figure legends, similar trends were consistently observed in all three donors. Complete datasets are available as S1 File.

## Results

### Experimental study models

**Fig 1:**
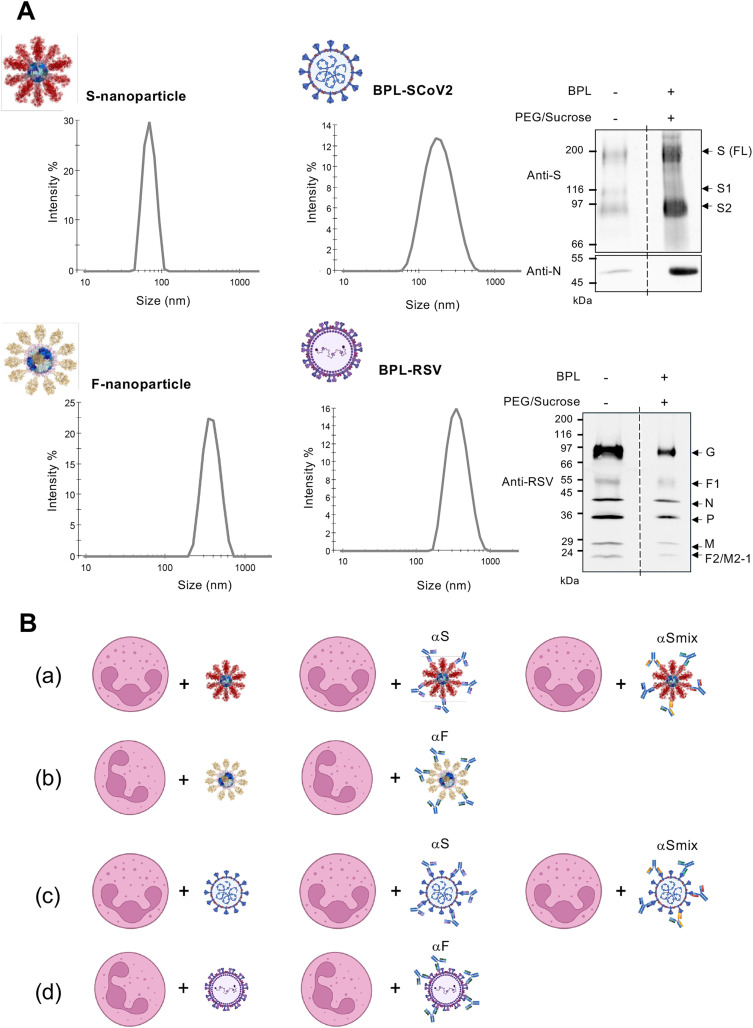
Experimental models. (A) Self-assembling nanoparticles displaying prefusion-stabilized trimeric SARS-CoV-2 Spike (S) or RSV Fusion (F) glycoproteins, as described in *Methods,* were analyzed by dynamic light scattering (DLS) for particle characterization. Purified β-propiolactone (BPL)-inactivated SARS-CoV-2 (SCoV2, Wuhan strain) and RSV A2 were assessed by immunoblot for viral proteins (S, N, or RSV antigens; Complete blots in S2 File) and by dynamic light scattering (DLS). (B) *Ex vivo* models used in this study consisted of incubating human neutrophils with S-nanoparticles or BPL-SCoV2, either alone or pre-coated with anti-S antibodies—either a single monoclonal (αS) or a mix of five monoclonals (αSmix), as detailed in **Table 1**. Parallel experiments were conducted using F-nanoparticles or BPL-inactivated RSV A2.

To investigate the influence of the SARS-CoV-2 S protein on human neutrophils *ex vivo*, we employed two distinct experimental systems (**Fig 1**). First, we utilized immunogenic nanoparticles displaying the 2P-stabilized prefusion trimeric SARS-CoV-2 S glycoprotein (LuS-N71-SpyLinked-CoV-2 S nanoparticles, hereafter referred to as S-nanoparticles). As a comparator to determine whether the observed effects were specific to SARS-CoV-2 S or a more general feature of respiratory virus glycoproteins exposed on nanoparticles, we used nanoparticles presenting DS2 stabilized prefusion trimeric respiratory syncytial virus (RSV) fusion (F) glycoproteins (LuS-N71-SpyLinked-RSV F nanoparticles, hereafter referred to as F-nanoparticles). The RSV prefusion F protein is a well-characterized respiratory virus antigen that was used in Arexvy, (GlaxoSmithKline) and Abrysvo (Pfizer) vaccines and successfully elicited protective immune responses [50,51]. The production of S- and F-nanoparticles has been previously described [35]. These nanoparticles present trimeric glycoproteins in a multivalent format and have been shown to induce significantly stronger neutralizing responses compared to recombinant S protein alone [35]. Therefore, the nanoparticle platform provides a valuable tool for studying the impact of the SARS-CoV-2 S protein in the prefusion trimeric state on neutrophil activation. Characterization of the S- and F-nanoparticle preparations by dynamic light scattering (DLS) revealed a single peak at the expected size ( [35], **Fig 1A**), confirming their homogeneity and structural integrity at the time of use.

Second, to assess the impact of the SARS-CoV-2 S protein within the structural context of a virus – and in a format relevant to inactivated virus vaccines – we used β-propiolactone (BPL)-inactivated, purified virus preparations. BPL-mediated inactivation is a widely used approach, notably employed in the CoronaVac (Sinovac) vaccine against SARS-CoV-2 [52]. Immunoblot analysis of purified, inactivated SARS-CoV-2 and RSV confirmed the presence of viral proteins (**Fig 1A**). Consistent with previous reports, BPL-inactivated SARS-CoV-2 virions exhibited a mix of prefusion and postfusion (mostly S2) peptides [53]. DLS analysis confirmed the uniformity and structural integrity of the virions, revealing single peaks at approximately 200 nm for SARS-CoV-2 and 300–400 nm for RSV (**Fig 1A**). Virus preparations exhibited excellent stability as per DLS measurements when stored at 4°C for at least 48 hours post-thaw. Thus, all subsequent experiments were conducted using thawed virion preparations stored at 4°C within this timeframe.

### Impact of trimeric prefusion SARS-CoV-2 S on human neutrophils *ex vivo*

To assess the impact of the prefusion trimeric SARS-CoV-2 S glycoprotein on neutrophils, we utilized an *ex vivo* system in which primary human neutrophils were exposed to S-nanoparticles and for comparison to F-nanoparticles (**Fig 1B**). Neutrophil viability, surface expression of adhesion, degranulation, IgG interactions and complement activation and regulation markers, ROS production, NET formation, and cytokine levels were systematically measured.

**Fig 2:**
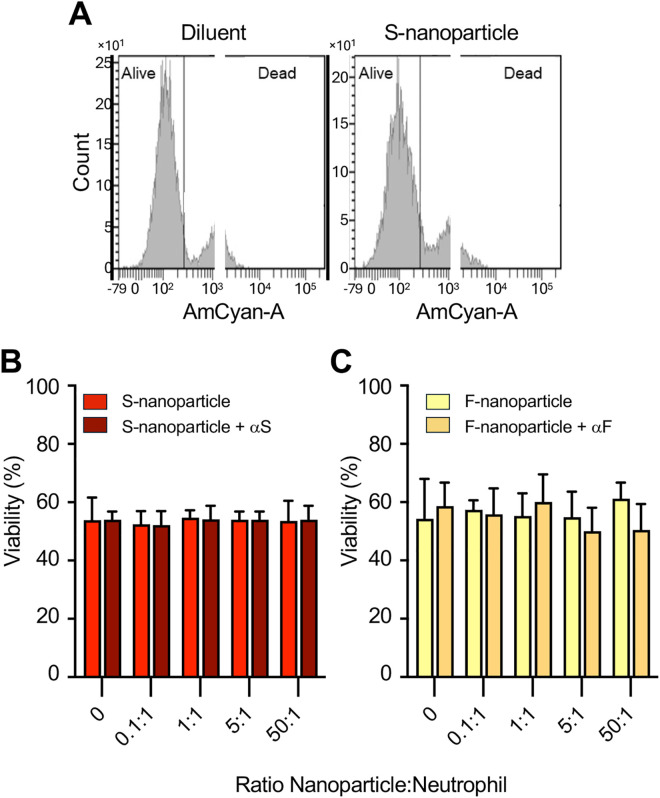
Effect of S-nanoparticles on neutrophil viability. (A) Human neutrophils were incubated for 24 h with S-nanoparticles or diluent control. Viability was assessed based on membrane integrity, as described in *Methods*. A representative experiment is shown. (B-C) Neutrophils were incubated for 24 h with the indicated S- (B) or F-nanoparticle (C)-to-neutrophil ratios, either alone or pre-coated with their respective antibodies (αS or αF, as in **Fig 1**). Data show mean ± SEM from three independent experiments, each using neutrophils from a different donor. Statistical analyses were performed using a two-way RM ANOVA.

Neutrophil viability was assessed after 24 h of exposure to increasing nanoparticle-to-neutrophil ratios, as described in *Methods*. Regardless of the S-nanoparticle concentration, approximately 55% of neutrophils remained viable, comparable to control conditions (**Fig 2A,B**). Similarly, F-nanoparticles protein had no discernible effect on neutrophil viability, further supporting the lack of cytotoxic impact (**Fig 2C**).

**Fig 3:**
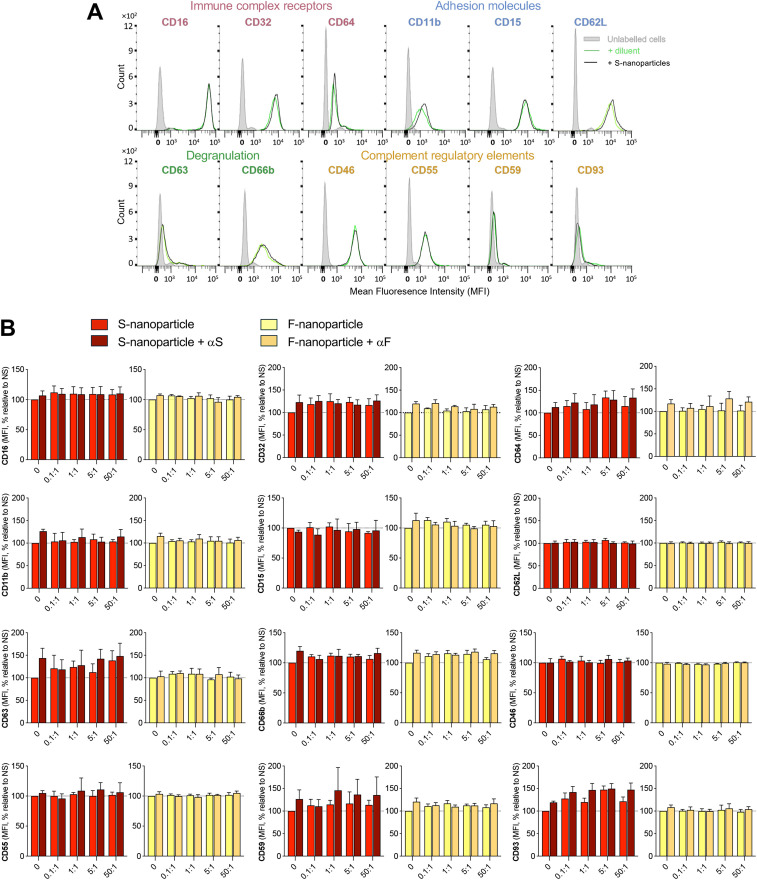
Impact of S-nanoparticles alone or pre-coated with anti-S antibodies on neutrophil surface marker expression. Neutrophils were incubated for 30 min or 3 h with the indicated S-nanoparticle-to-cell ratios, either alone or pre-coated with a monoclonal anti-S antibody (αS; see **Fig 1**). F-nanoparticles, with or without pre-coating with anti-F antibody (αF; see **Fig 1**), were used as comparators. Surface markers were stained with fluorophore-conjugated antibodies and analyzed by flow cytometry (**Table 2**). Markers were grouped by function: immune complex receptors (CD16, CD32, CD64), adhesion (CD11b, CD15, CD62L), degranulation (CD63, CD66b), and complement regulation (CD46, CD55, CD59, CD93). (A) A representative experiment using a S-nanoparticle-to-cell ratio of 50:1 incubated for 30 min is shown. (B) Quantified data from 30 min incubations are shown as mean ± SEM (n = 3 donors). Surface expression is reported as percent change in mean fluorescence intensity (MFI) relative to non-stimulated (NS) cells. Full results, including 3 h data, are presented in S1 Table.

Next, we examined a panel of neutrophil surface markers associated with adhesion (CD11b, CD15, CD62L), degranulation (CD63, CD66b), IgG interactions (CD16, CD32, CD64), and complement activation and regulation (CD46, CD55, CD59, CD93). Neutrophils were incubated with increasing nanoparticle-to-neutrophil ratios of S-nanoparticles for 30 min (**Fig 3**) or 3 h (S1 Table). Flow cytometry analysis, which we previously reported allows to detect variation in surface markers expression [44], revealed that S-nanoparticles had no significant impact on the expression of any of the quantified markers at any of the ratio tested (**Fig 3A**-B and S1 Table). These results are similar to surface markers expression observed when neutrophils were incubated with F-nanoparticles (**Fig 3B** and S1 Table).

**Fig 4:**
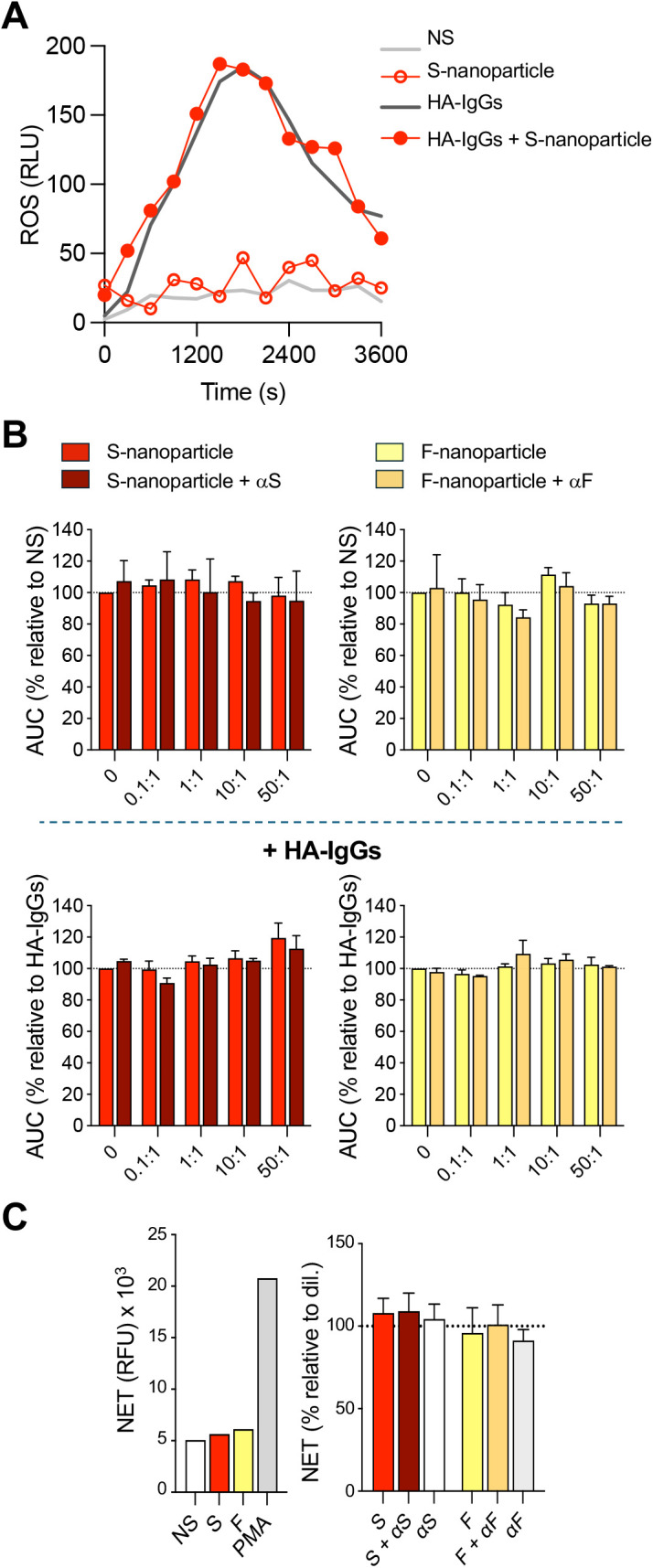
Effect of S-nanoparticles on neutrophil ROS production and NET formation. Human neutrophils were left unstimulated (NS) or incubated with the indicated S-nanoparticle-to-cell ratios, either alone or in the presence of 1 mg/mL heat-aggregated immunoglobulins (HA-IgGs). S-nanoparticles were used either uncoated or pre-coated with anti-S antibody (αS; **Fig 1**). F-nanoparticles, with or without anti-F antibody (αF, **Fig 1**), were included as comparators. (A–B) ROS production was monitored over 60 min by luminol-based chemiluminescence. (A) A representative experiment using a nanoparticle-to-neutrophil ratio of 50:1 is shown; results are expressed as relative luminescence units (RLU). (B) ROS levels are quantified as area under the curve (AUC) and expressed as percentages relative to NS or HA-IgG-stimulated cells, as indicated. (C) NET formation was measured after 4 h of incubation at a nanoparticle-to-neutrophil ratio of 50:1 using SYTOX green staining, as described in *Methods*. PMA (10 nM) was used as a positive control. Left: representative experiment, expressed as relative fluorescence units (RFU). Right: NET production expressed as percentage relative to NS. Data in (B) and (C) are shown as mean ± SEM from n = 3 independent experiments with neutrophils from different donors.

As key players in the innate immune response, neutrophils generate ROS and NETs in response to various stimuli. Elevated ROS levels correlate with neutrophil counts in patients with severe COVID-19 [54], while excessive NETosis has been associated with disease severity [14–16]. However, the role of the prefusion trimeric S glycoprotein in these processes remains unknown. To assess whether S-nanoparticles could induce ROS production or modulate neutrophil ROS responses to established stimuli, we measured ROS levels following S-nanoparticle exposure in the presence or absence of heat-aggregated (HA)-IgGs, a model for immune complexes [55]. S-nanoparticles had no discernible impact on ROS production, either alone or in combination with HA-IgGs, a finding that mirrored results observed with F-nanoparticles (**Fig 4A-B**). We next investigated whether S-nanoparticles could directly induce NET formation. Unlike PMA, a well-established NETosis inducer, S-nanoparticles had no significant effect on NET production. F-nanoparticles similarly failed to induce NETosis (**Fig 4C**).

**Fig 5:**
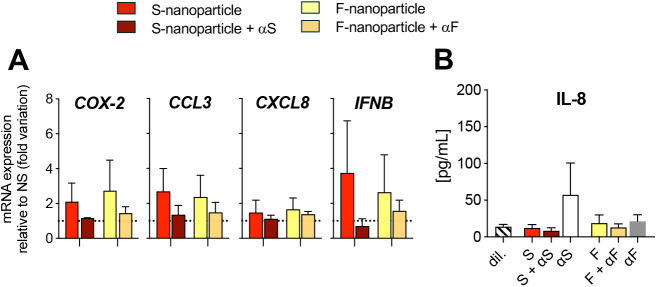
Effect of S-nanoparticles on inflammatory gene expression and cytokine release. Human neutrophils were incubated for 60 min with S- or F-nanoparticles at a nanoparticle-to-cell ratio of 50:1, either uncoated or pre-coated with their respective antibodies (**Fig 1**). (A) mRNA levels of the indicated genes were quantified by RT-qPCR, normalized to GAPDH, and expressed as fold change relative to non-stimulated cells (NS). *Non-significant increases in COX-2, CCL3, and IFNB mRNA were observed, driven by responses in subsets of donors.* (B) IL-8 levels in the supernatant were quantified using multiplexed bead-based immunoassay. All data represent mean ± SEM from n = 3 independent experiments using neutrophils from different donors.

Cytokine storm is a hallmark of COVID-19 complications [3], and neutrophils are thought to contribute to the excessive inflammation. To investigate the inflammatory gene response, we exposed neutrophils to S-nanoparticles or F-nanoparticles for 60 min and measured the expression of genes encoding COX-2, CCL3 (MIP-1α), CXCL8 (IL-8), and IFNβ. A trend toward increased *COX-2*, *CCL3*, and *IFNB* mRNA was observed in nanoparticles-exposed neutrophils, though this was not statistically significant and was driven mainly by responses in a subset of donors (COX-2 and IFNB in 1/3, CCL3 in 2/3) (**Fig 5A**). We also examined cytokine secretion by measuring IL-1α, IL-1β, IL-6, CXCL8 (IL-8), TNF, IFNα, CCL3 (MIP-1α), and CCL2 (MCP-1) in the supernatants from neutrophils exposed to nanoparticles for 4 h. Of these, only IL-8 was detected. Neither S- nor F-nanoparticles affected IL-8 levels of cytokines (**Fig 5B**).

### Inactivated SARS-CoV-2 has minimal impact on neutrophil responses

**Fig 6:**
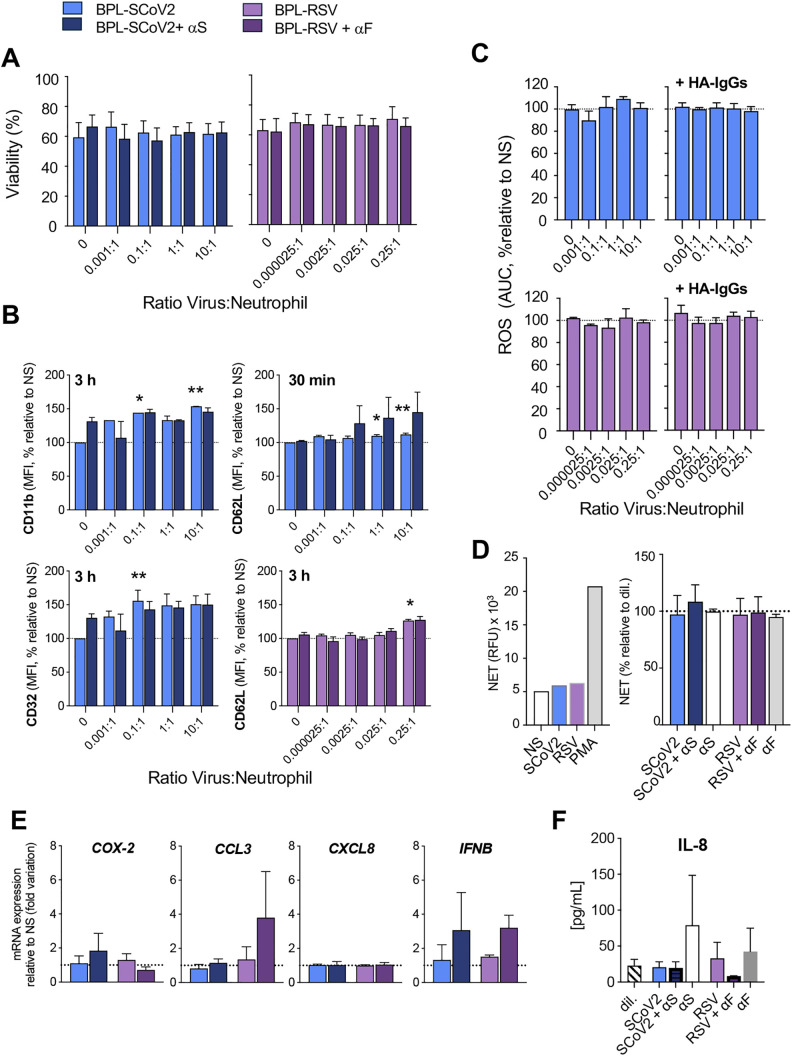
Effect of inactivated SARS-CoV-2 on neutrophil responses. Human neutrophils were incubated with the indicated ratios of BPL-inactivated SARS-CoV-2 (SCoV2), either uncoated or pre-coated with anti-S antibody (αS). BPL-inactivated RSV, with or without anti-F antibody (αF), was used as a comparator (**Fig 1**). (A) Neutrophil viability was assessed after 24 h, as described in **Fig 2**. (B) Surface expression of markers related to immune complex receptors (CD16, CD32, CD64), adhesion (CD11b, CD15, CD62L), degranulation (CD63, CD66b), and complement regulation (CD46, CD55, CD59, CD93) was measured by flow cytometry after 30 min and 3 h of incubation, as in **Fig 3**. Shown are markers (CD11b, CD32 and CD62L) exhibiting significant changes; full results are available in S2 Table. Data are expressed as percent change in mean fluorescence intensity (MFI) relative to non-stimulated (NS) cells. (C) ROS production was measured in the absence or presence of HA-IgGs, as in **Fig 4**. Results are expressed as area under the curve (AUC), relative to NS or HA-IgG-stimulated cells. (D) NET formation was quantified after 4 h, as in **Fig 4**. Left: A representative experiment using a virus-to-neutrophil ratio of 10:1 is shown. PMA (10 nM) was used as a positive control; results are expressed as relative luminescence units (RLU). Right: NET production expressed as percentage relative to NS. (E) mRNA levels of selected inflammatory genes were measured by RT-qPCR, as described in **Fig 5**. IL-8 levels in the supernatant were quantified using multiplexed bead-based immunoassay. All data represent mean ± SEM from n = 3 independent experiments using neutrophils from different donors. * *P* < 0.05; ** *P* < 0.01.

Results obtained with S-nanoparticles provided no evidence that the trimeric prefusion-stabilized S protein significantly influences neutrophil responses. To investigate whether the S protein in the context of a viral structure – relevant to inactivated virus vaccines – elicits different effects, we examined the impact of the purified BPL-inactivated SARS-CoV-2 in the *ex vivo* system with primary human neutrophils (**Fig 1B**). For comparison, we also used BPL-inactivated RSV. Neutrophil viability remained unaffected following 24 h exposure to increasing ratios of inactivated SARS-CoV-2 or RSV to neutrophils (**Fig 6A**). Expression of most surface markers associated with degranulation, IgG interactions, complement activation and regulation was largely unchanged after 30 min or 3 h exposure to inactivated SARS-CoV-2 (**Fig 6B and** S2 Table). However, exposure to inactivated SARS-CoV-2 led to an upregulation of CD11b—and to a lesser extent, CD62L—both of which are associated with cell adhesion. This effect was observed after 3 h and 30 min of exposure, respectively. Induction of CD62L was also observed after exposure to inactivated RSV for 3 h. An increasing trend of CD32 expression, associated with IgG interactions, was also observed after 3 h of exposure to inactivated SARS-CoV-2 (**Fig 6B and** S2 Table). Inactivated SARS-CoV-2 had no influence on ROS production (**Fig 6C**) in the absence or presence of HA-IgGs, NET formation (**Fig 6D**), inflammatory gene expression (**Fig 6E**), or IL-8 release (**Fig 6F**) at any of the virus particle-to-neutrophil ratio tested. These results are similar to neutrophil treatment with inactivated RSV. Collectively, these findings reinforce the conclusion that the SARS-CoV-2 S protein, even in the context of an inactivated virus, has little to no effect on the tested neutrophil responses.

### Antibody binding to Spike does not alter its effect on neutrophil responses

A diverse array of anti-S antibodies is generated following SARS-CoV-2 infection or vaccination [56]. Consequently, upon subsequent encounters, the S glycoprotein is likely to be antibody-bound, facilitating recognition by phagocytes, including neutrophils. To assess the effect of antibody-bound S glycoprotein on neutrophils in the *ex vivo* system, we first pre-coated S-nanoparticles with a single neutralizing monoclonal anti-S antibody (αS, **Table 1 and Fig 1B**). This antibody, derived from B cells specific for the SARS-CoV-2 S glycoprotein, was isolated from a COVID-19-infected individual and targets the receptor-binding domain (RBD; residues 319–591), effectively blocking the RBD-ACE2 interaction [57]. For comparison, we pre-coated F-nanoparticles with Palivizumab, a clinically approved humanized monoclonal antibody (αF; **Table 1 and Fig 1B**) used to prevent severe lower respiratory tract infections (LRTI) caused by RSV. Palivizumab prevents virus entry by inhibiting viral-host membrane fusion and may also suppress cell-to-cell transmission by blocking syncytia formation in respiratory epithelial cells. As a result, it reduces RSV virulence and the risk of RSV-related LRTI [58]. In this experimental setting, antibody-bound S- and F-nanoparticles had no significant effect on neutrophil viability (**Fig 2B**), surface marker expression (**Fig 3B** and S1 Table), ROS production (**Fig 4B**), NET formation (**Fig 4C**), inflammatory gene expression (**Fig 5A**), or cytokine secretion (**Fig 5B**). Similarly, neutrophil viability (**Fig 6A**), ROS production (**Fig 6C**), NETosis (**Fig 6D**), and IL-8 release were unaffected by inactivated SARS-CoV-2 pre-coated with αS. Amongst surface marker expression, a non-significant trend toward increased CD62L expression was observed in response to antibody-coated SARS-CoV-2 (**Fig 6B and** S2 Table). This trend was not observed with αF-coated inactivated RSV (**Fig 6B and** S2 Table). Similarly, antibody-coated inactivated SARS-CoV-2 and RSV were associated with a non-significant trend toward increased *IFNB* gene expression (**Fig 6E**).

**Fig 7:**
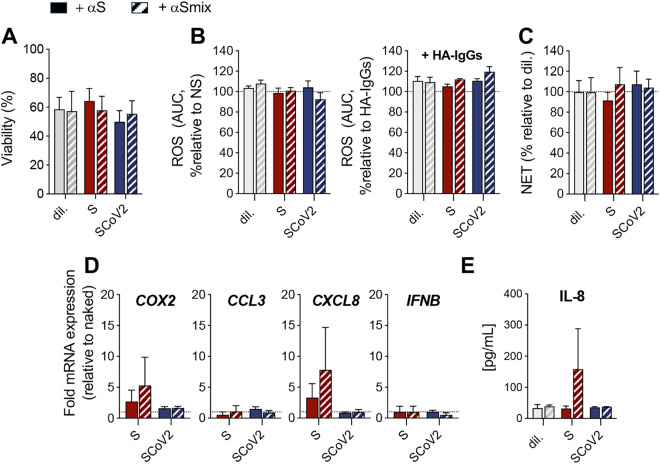
Comparison of single vs multiple anti-Spike antibodies for nanoparticle and virus pre-coating on neutrophil responses. Neutrophils were incubated with diluent (dil), S-nanoparticles, or BPL-inactivated SARS-CoV-2 (SCoV2), either pre-coated with a single monoclonal anti-S antibody (αS) or with a mixture of five monoclonal anti-S antibodies (αSmix), as described in **Fig 1**. Neutrophil viability (A), ROS production (B), NET formation (C), inflammatory gene expression (D), and IL-8 release (E) were assessed and analyzed as described in Figs 2**–**5. Surface marker expression data are presented in S3 Table. A non-significant trend toward increased *COX2* and *CXCL8* gene expression and IL-8 release was noticed. All results are shown as mean ± SEM from n = 3 independent experiments, each performed with neutrophils from a different donor.

Since natural and vaccine-induced immune responses generate polyclonal antibodies against multiple S epitopes, we next examined whether a diverse antibody mix altered neutrophil responses differently than a single neutralizing antibody. This approach provides a more physiologically relevant model of immune complex formation and FcγR-mediated neutrophil activation. S-nanoparticles or BPL-inactivated SARS-CoV-2 were pre-coated with a mix of five anti-S antibodies (αSmix; **Table 1 and Fig 1B**) before exposure to neutrophils. Coating with the αSmix did not impact neutrophil viability, surface marker expression, ROS and NET production compared to coating with αS (**Fig 7 and** S3 Table). However, a non-significant trend toward increased *COX2* and *CXCL8* gene expression (**Fig 7D**) and IL-8 release (**Fig 7E**) was noticed.

Collectively, these findings indicate that antibody binding does not significantly alter the neutrophil response to the S protein, whether using a single neutralizing antibody or a diverse antibody mix. These results suggest that FcγR engagement by Spike-containing immune complexes is insufficient to drive substantial neutrophil activation under these conditions.

## Discussion

The SARS-CoV-2 S protein is essential for viral entry and is the principal target of neutralizing antibodies, making it a key component in COVID-19 vaccines. Understanding how the S protein interacts with innate immune cells, especially neutrophils, is critical given their dual role in antiviral defense and inflammatory pathology. In this study, we evaluated whether the prefusion-stabilized trimeric S protein directly induces neutrophil responses *ex vivo*. Using two biologically relevant models—nanoparticle-displayed S and BPL-inactivated SARS-CoV-2—we found no evidence that S alone, in these contexts, elicits robust neutrophil responses.

Our models were selected to reflect relevant vaccine contexts. The S-nanoparticles mimic the multivalent display of prefusion S found on intact virions, recombinant protein nanoparticle vaccines (e.g., Novavax), and membrane-anchored S expressed on host cells following mRNA vaccination. The BPL-inactivated virus, similar to the CoronaVac vaccine, presents a mixture of prefusion and postfusion Spike conformations within the native virion structure, as previously reported [53]. These models therefore encompass different physical presentations and contexts of the S protein relevant to both infection and vaccination.

Most previous studies reporting S-mediated neutrophil activation employed soluble recombinant S protein, which differs markedly from the multivalent, trimeric, and membrane-bound or particle-anchored forms used here. This difference in presentation is significant, as the conformation, oligomerization state, and valency of the S protein are likely to influence receptor engagement and downstream immune activation. Supporting the relevance of our model, the same S-nanoparticles used in this study have previously elicited potent neutralizing responses—25-fold higher than trimeric soluble S protein—in a mouse immunization model at low antigen doses [35]. Despite this immunogenicity, we found that S-nanoparticles induced little to no neutrophil responses under the tested conditions. Across a range of nanoparticle-to-neutrophil ratios, we observed no significant modulation of surface markers reflecting adhesion, degranulation, IgG interactions and complement activation and regulation, ROS production, NETosis, inflammatory gene expression or cytokine release. In line with our findings, prior reports have produced conflicting evidence regarding neutrophil activation by recombinant S. One study found that full-length recombinant S, but not S1 or S2 subunits, could induce NETs in a ROS-independent manner at high S:neutrophil ratios [59,60], while another observed increased ROS and IL-8/IL1-Ra in response to recombinant S1 protein [61]. In contrast, Ait-Belkacem et al. reported that trimeric prefusion S induced only minimal granulocyte responses in whole blood, with no significant changes in any neutrophil activation markers and only a trend toward higher CD64 expression [62]. Additionally, Veras et al. demonstrated that live, replicating SARS-CoV-2 is required for NETosis induction, suggesting that S alone may be insufficient [14]. This aligns with our observation that BPL-inactivated SARS-CoV-2 failed to induce detectable neutrophil activation.

Glycosylation is another critical determinant of S immunogenicity and receptor interactions. The glycan composition of S protein can vary significantly between expression systems, affecting how immune cells recognize and respond to it. The SARS-CoV-2 S protein contains over 20 N-linked glycosylation sites, which influence epitope masking and structural stability [63]. Studies show that removing the glycan shield enhances immunogenicity and protection [64]. The impact of BPL inactivation on S glycosylation is not fully characterized, though the efficacy of CoronaVac suggests preserved recognition by immune cells. Similarly, S-nanoparticles were produced in a mammalian expression system designed to generate glycosylated proteins with appropriate immunogenic features. While subtle glycosylation differences may exist, the potent immune responses observed in prior mouse studies argue against a major impairment in antigenicity or neutrophil recognition [35].

In this study, we evaluated S from the Wuhan strain in the BPL-inactivated virus and the D614G variant in the S-nanoparticles model. Since S mutations can modulate ACE2 binding, immune escape, and antigenicity, it remains possible that variant-specific differences influence neutrophil responses. For instance, the Omicron variant carries mutations that alter both antibody binding and innate immune engagement. Therefore, we cannot exclude that different results would be obtained with S protein from different variants.

Although our *ex vivo* system provides a valuable platform to assess direct neutrophil responses to prefusion multivalent trimeric S protein, it does not fully replicate the complex tissue environment encountered *in vivo*. Particularly, it lacks the cellular complexity, such as interaction with vascular endothelial cells and other immune cells, and inflammatory cues, including DAMPs or inflammatory cytokines (e.g., IL-1β or GM-CSF). It is conceivable that during infection or following vaccination, primed neutrophils may respond differently to the S protein. In this study, we did model one such context by pre-coating S-nanoparticles with anti-S antibodies, mimicking the formation of immune complexes following infection or vaccination. Because antibody glycosylation critically shapes effector function, the IgGs used in this study were produced in HEK293 cells to yield glycosylation profiles closely resembling those of native human antibodies, though not identical [42]. Even under these conditions, we found no evidence of enhanced neutrophil responses via Fcγ receptor engagement, either with a neutralizing monoclonal antibody or a mixture of monoclonals targeting diverse S epitopes. These data suggest that immune complex formation alone may be insufficient to trigger neutrophil responses in the absence of additional inflammatory or danger signals. Furthermore, the absence of activation under the experimental conditions used in this study does not exclude that there could be an indirect role for the S protein in the recruitment or priming of neutrophils *in vivo*. Future studies incorporating other cofactors or using *in vivo* models will be essential to further define the role of the S protein in neutrophil activation.

## Supporting information

S1 Table Impact of S-nanoparticles and F-nanoparticles alone or pre-coated with antibodies on neutrophil surface marker expression. (DOCX)

S2 Table Impact of inactivated-SARS-CoV-2 and -RSV alone or pre-coated with antibodies on neutrophil surface marker expression. (DOCX)

S3 Table Impact of pre-coating of nanoparticles or inactivated virus with a single antibody vs a mix of five antibodies on neutrophil surface marker expression. (DOCX)

S1 File Full datasets use to generate graphs and tables presented in the study. (XLSX)

S2 File Original blots for Figure 1A. (PDF)
