# Supplementary material for: No evidence of direct activation of human neutrophil responses by multivalent prefusion trimeric SARS-CoV-2 Spike protein ex vivo
Source: PLoS One. 2025 Oct 29;20(10):e0332261. doi: 10.1371/journal.pone.0332261 (PMC12571262; doi:10.1371/journal.pone.0332261)
Supplement: S1 Table — (DOCX) [file pone.0332261.s001.docx]

**A)**

**B)**

**C)**

**D)**

**Table S1**. **Impact of S-nanoparticles and F-nanoparticles alone or pre-coated with antibodies on neutrophil surface marker expression.** Neutrophils were incubated for 30 min or 3 h with the indicated S-nanoparticle-to-cell ratios, either alone or pre-coated with a monoclonal anti-S antibody (αS; **Table 1**). F-nanoparticles, with or without pre-coating with anti-F antibody (αF; **Table 1**), were used as comparators. Surface markers were stained with fluorophore-conjugated antibodies and analyzed by flow cytometry (**Table 2**). Markers were selected for the monitoring of the following parameters: (**A)** Interactions with immune complexes (CD16, CD32, CD64); (**B)** Adhesion (CD11b, CD15, CD62L); (**C)** Degranulation of primary (CD63) and secondary (CD66b) granules; and (**D)** Complement regulation (CD46, CD55, CD59, CD93). Results were expressed as percent change in the mean fluorescence intensity (MFI) relative to non-stimulated cells. Data are shown as mean ± SEM, n=3; except CD11b & CD93; S & S + αS, 30 min & 3 hrs: mean ± SD, n=2; (Complete dataset **S2 File**). n.s.: non-significant. No significant impact on the expression of the quantified markers was observed at any of the tested ratios and in the presence or absence of coating with antibodies.
