# Supplementary material for: No evidence of direct activation of human neutrophil responses by multivalent prefusion trimeric SARS-CoV-2 Spike protein ex vivo
Source: PLoS One. 2025 Oct 29;20(10):e0332261. doi: 10.1371/journal.pone.0332261 (PMC12571262; doi:10.1371/journal.pone.0332261)
Supplement: S2 Table — (DOCX) [file pone.0332261.s002.docx]

**A)**

**B)**

**C)**

**D)**

**Table S2**. **Impact of Inactivated-SARS-CoV-2 and -RSV alone or pre-coated with antibodies on neutrophil surface marker expression.** Neutrophils were incubated for 30 min or 3 h with the indicated BPL-inactivated SARS-CoV-2 (SCoV2)-to-cell ratios, either alone or pre-coated with a monoclonal anti-S antibody (αS; **Table 1**). BPL-inactivated RSV, with or without pre-coating with anti-F antibody (αF; **Table 1**), were used as comparators. Surface markers were stained with fluorophore-conjugated antibodies and analyzed by flow cytometry (**Table 2**). Markers were selected for the monitoring of the following parameters: (**A)** Interactions with immune complexes (CD16, CD32, CD64); (**B)** Adhesion (CD11b, CD15, CD62L); (**C)** Degranulation of primary (CD63) and secondary (CD66b) granules; and (**D)** Complement regulation (CD46, CD55, CD59, CD93). Results were expressed as percent change in the mean fluorescence intensity (MFI) relative to non-stimulated cells. Data are shown as mean ± SEM, n = 3 donors; except for CD64; RSV; n=2 ± SD (Complete dataset **S2 File**). When statistically different from non-stimulated cells, *P* values are indicated in Bold. n.s.: non-significant. Inactivated SARS-CoV-2 induced CD11b (3 h) and CD62L (30 min) upregulation. An increase was observed with a modest increase in CD32 (3 h), while most other markers remained unchanged. CD62L induction was also observed following 3 h exposure to inactivated RSV. Antibody-coated SARS-CoV-2 induced a non-significant trend toward increased CD62L expression, which was not observed with αF-coated RSV.
