# Supplementary material for: No evidence of direct activation of human neutrophil responses by multivalent prefusion trimeric SARS-CoV-2 Spike protein ex vivo
Source: PLoS One. 2025 Oct 29;20(10):e0332261. doi: 10.1371/journal.pone.0332261 (PMC12571262; doi:10.1371/journal.pone.0332261)
Supplement: S3 Table — (DOCX) [file pone.0332261.s003.docx]

**Table S3**. **Impact of pre-coating of nanoparticles or inactivated virus with a single antibody vs a mix of five antibodies on neutrophil surface marker expression.** Neutrophils were incubated for 30 min or 3 h with S-nanoparticles (S; ratio S-nanoparticle : neutrophil = 50:1) or BPL-inactivated SARS-CoV-2 (SCoV2; ration SCoV2 : neutrophil = 10:1) either alone or pre-coated with single a monoclonal anti-S antibody (αS; **Table 1**) or a mixture of five monoclonal antibodies (αSmix; **Table 1**). The indicated surface markers were stained with fluorophore-conjugated antibodies and analyzed by flow cytometry (**Table 2**). Results were expressed as percent change in the mean fluorescence intensity (MFI) relative to cells incubated with antibodies alone. Data are shown as mean ± SEM, n = 3; except for CD64: mean ± SD, n=1 (Complete dataset **S2 File**). n.s.: non-significant. Coating with the αSmix did not impact surface marker expression compared to coating with αS.
