# Supplementary figures and images for: No evidence of direct activation of human neutrophil responses by multivalent prefusion trimeric SARS-CoV-2 Spike protein ex vivo
Source: PLoS One. 2025 Oct 29;20(10):e0332261. doi: 10.1371/journal.pone.0332261 (PMC12571262; doi:10.1371/journal.pone.0332261)

Original blots Fig. 1A

Anti-S

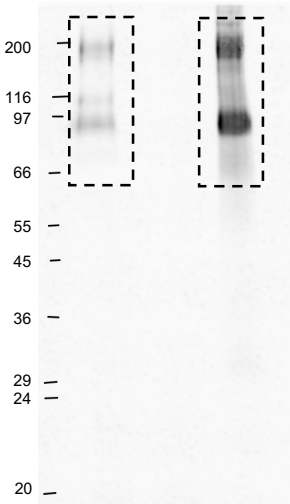

Anti-N

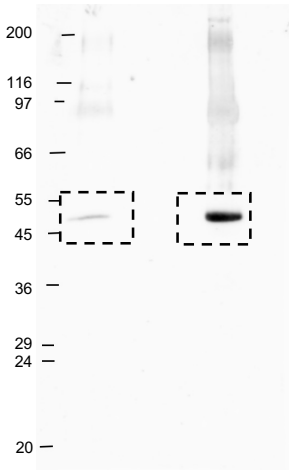

Anti-RSV

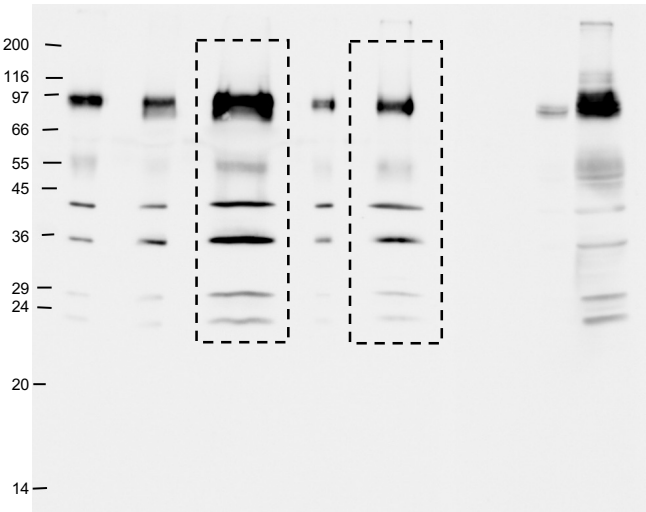

Supplement: S2 File — (PDF) [file pone.0332261.s005.pdf]
